# Cottage Hospital Ward Walls

**Published:** 1889-12-28

**Authors:** 


### THE WALLS OF COTTAGE HOSPITAL WARDS.

"F.R.C.S." writes: "May I be allowed through the medium of your columns to seek information up to date as to the best materials for painting and colouring the walls of a cottage hospital—a new building erected two years ago, and now ready for internal decoration? In particular, it is desired to know how far the promises (if any) of the "distempers" and "sanitary paints" are justified by actual performance. Any information, editorial or otherwise, will be thankfully received."

*** The best way to treat the walls of a cottage hospital is to paint from the floor upwards to a height of about five to six feet, and then to varnish the painted surface with two coats of the best copal varnish. Above this the walls should be distempered. The use of a washable distemper is not to be commended, its chief value being that it is more durable than ordinary distemper. Durability is not an object to be sought for in the covering of a ward wall, unless it can at the same time be made impervious. The walls of a ward should be periodically washed, and the distemper removed entirely so that the bare plaster is exposed. The adoption of a washable distemper would lead to this being done less frequently, while the surface would be no less impervious than ordinary distemper.

The periodical and systematic scraping and cleansing of the wall surface is of the utmost hygienic value, and it may even be doubted if it is exceeded in value by the most impervious wall surface which can be had. As regards paint there is nothing of special importance to be said. Everyone nowadays knows the necessity for avoiding the use of mineral greens. If zinc white could be used as a base instead of white lead it would be an advantage, but the benefit would be to the workmen who apply the paint, not to the inmates of the hospital.

